# How Do We Calibrate a Battery Electric Vehicle Model Based on Controller Area Network Bus Data?

**DOI:** 10.3390/s24144637

**Published:** 2024-07-17

**Authors:** Dávid Tollner, Ádám Nyerges, Mahmoud Said Jneid, Attila Geleta, Máté Zöldy

**Affiliations:** 1 Department of Automotive Technologies, Budapest University of Technology and Economics, 1111 Budapest, Hungary; nyerges.adam@kjk.bme.hu (Á.N.); mahmoud.jneid@kjk.bme.hu (M.S.J.); 2 Robert Bosch Kft., 1103 Budapest, Hungary; attila.geleta@hu.bosch.com

**Keywords:** data logging, simulation, electrical consumption, electric vehicles

## Abstract

Transforming an up-to-date vehicle into a measurement system is a rewarding task due to the large number of different sensors in the onboard control and diagnostic systems. These procedures are not performed by a single control unit; it is necessary to share the signal values over a communication network, to which an external device can be connected to record the real traffic. The paper aims to use these recorded data for 1 DOF longitudinal vehicle and powertrain model validation. For repeatability, three city routes are selected: plain road, smaller road grade, and higher road grade in both directions. Therefore, the drivetrain system is tested in a high load range, even with long-term recuperation. The altitude changes are recorded with a DGPS system. By the recorded measurements, the vehicle and the drivetrain model can be calibrated, such as the air drag parameters, the rolling resistances, and the efficiencies of the drivetrain. The validation criteria are defined for speed tracking, and the relative tolerance of the cumulated energy should be below 10%. At the end of the day, a developed model is ready for energetic analysis or control strategy design. The energy balance of the applied cycles is also presented to prove that.

## 1. Introduction

In recent years, sustainability has strongly influenced the automotive industry and mobility in design and manufacturing processes. Tighter emission standards are supporting the development of alternative fuel powertrains [1]. The electric powertrain control module had to process a large number of sensor values simultaneously, such as the voltage levels of the cells in the high-voltage (HV) battery and the inverter and motor temperatures, to ensure that they were within the appropriate range before damage or overheating occurred. These can be prevented by reducing power. Measuring stator temperature is typically challenging, as the sensor only measures the temperature at one point. The temperature can be determined based on a model for the rest of the surface. Determining the temperature of the rotor is an even more complicated problem because of its rotating motion. Furthermore, the actual State of Charge (SoC) and State of Health (SoH) of the HV battery is also a complex task, usually based on a battery model [2,3,4,5].

The inputs to these models are quantities measured by different sensors. An electric car powertrain needs 1 or 2 speed and position sensors, 15–20 current sensors, and 20–30 temperature sensors, usually NTCs [6]. The values of these sensors are required by several electronic control units (ECUs); therefore, they can be found on the vehicle’s communication network. Based on these, it was obvious for us to log these data from the car for the simulation instead of installing new sensors in the vehicle.

Modeling and simulating the vehicle and drivetrain operation helps check the measured signals, estimate unmeasurable signals, and diagnose faults in the systems. It is also useful to improve the sampled and measured signals for better resolution.

Implementing battery electric vehicle (BEV) models is simpler than conventional drivetrains [7]. The lack of clutch and gear selection strategies makes the modeling process more accurate; it is easier to reproduce real-world driving [8].

Modern BEVs appeared less than two decades ago, and their development is a very focused area even today. The “optimal” BEV layout is not yet formed; there will be significant developments in efficiency, range, dynamics, and thermal management [9].

The paper will present a model that can be used for energetic analysis and control strategy development. The energy transformation is deeply understandable through model verification and validation, and the key development areas can be found.

## 2. Measurement Strategy

The powertrain of an electric car has significantly fewer components than an internal combustion version. However, much more information is available on the EVs’ in-vehicle communication network. There are several reasons for this: First, the number of ECUs in the vehicle continuously increases due to more advanced driver assistance systems and comfort functions [10]. Another reason is that in an electric car, the drive-related control systems must monitor many more parameters, from battery output current through inverter temperature to rotor position. It is also essential to maximize efficiency and lifetime, for example, by constantly monitoring the battery cell voltages to ensure that none of the cells are discharged below a threshold value, as this would permanently damage them. The development of ECUs has brought the Functional Safety Standard [11], which regulates the safe operation of safety-related electrical/electronic systems and broadly defines the hardware and software features of modern ECUs.

The complex monitoring system requires numerous sensors whose measured values can be retrieved from the vehicle’s communication network, as multiple ECUs typically require these. In most cases, the in-vehicle communication is unknown, so the data are only interpretable after complex reverse engineering. However, retrieving data from specific ECUs using a diagnostic protocol is possible.

### 2.1. Protocols

Automotive protocols can be divided into two main categories: communication and diagnostic protocols. The former defines the rules for transmitting information, such as the number of wires and voltage levels, while the latter defines the structure of the information packages, i.e., messages.

Despite the increased amount of in-vehicle information in EVs, the well-known Controller Area Network (CAN) 2.0 [12] protocol is generally used by car manufacturers for communication between ECUs due to its low cost and simplicity. However, some Advanced Driver-Assistance Systems (ADAS) functions already require Automotive Ethernet [13] or FlexRay [14] for higher bandwidth.

An essential property of CAN is that it provides serial asynchronous data transmission. Therefore, the data recorded from the vehicle are very close in time (~50 ms), but there is still a time offset. This is a not negligible issue because if the data are not transformed to a shared time axis, there may be operating points where the vehicle speed is still increasing compared to the previous time point, but braking has already started.

The onboard diagnostic (OBD) standard [15] was the first official diagnostic standard, introduced in California in 1988. The purpose of this was to ensure that the vehicle constantly monitored the operation of the emission control systems. Later, the more specific OBD-II [16] standard was released, mandatory in the US since 1996 [17], for European petrol vehicles since 2001, and for European diesel vehicles since 2004 [18].

The OBD-II standard further includes that in the event of a malfunction or incorrect values, the vehicle must store the fault with the environmental parameters, which must be readable later. So, it has to support not only onboard but also off-board diagnostics, i.e., external devices must also be able to access the vehicle’s communication network. If an external device can be connected to the car, it seems evident that the values of the sensors monitored by the vehicle should be accessible to external devices. This enabled data logging.

The OBD-II diagnostic protocol is public so that the parameters can be recorded and tested in the same form on all vehicles. This means only general information can be checked, such as speed, engine speed, or throttle position. Due to the growth of hybrid cars, OBD-II has been extended to provide not only emission-relevant data, for example, the hybrid battery pack’s remaining life or the high-voltage battery SoC value.

For vehicle fault detection, not only the emission-relevant components are important, so the KeyWord Protocol 2000 (KWP) [19] was created as an extension of the OBD-II diagnostic standard, initially using the K-line bus system, and only a few ECUs were available with it. In the OBD-II standard, there was a connector [20] where SAE J1850 [21], K-line [22], and CAN bus systems are accessible, so KWP uses this connector as well.

KWP is not a generalized diagnostic, so it provides much more detailed data, such as steering wheel angle or brake system pressure. As a result, each vehicle has different ECU access addresses (identifiers) and values by which specific parameters can be accessed (local identifiers). Therefore, data logging is much more difficult with this protocol, as the software has to be parameterized for each vehicle.

The disadvantage of the K-line is that the maximum data rate is 10.4 Kbit/s, while with CAN, 1 Mbit/s is available. Therefore, the KWP2000 on CAN [23] standard has extended the diagnostic possibilities, such as flash programming of ECUs.

Unified diagnostic services (UDSs) [24] were created based on the KWP–CAN model to harmonize diagnostic standards. One of the advantages of UDS is that it is independent of the bus system, i.e., it works not only on CAN but also on Ethernet or FlexRay. Furthermore, in contrast to previous standards, periodic data requests are possible, so if continuous data logging is required, there is no need to send continuous data request messages to the ECUs.

### 2.2. Test Vehicle

The test vehicle was a 2011 Mitsubishi MiEV, produced from 2009 to 2021. The MiEV is one of the original first-generation electric vehicles, despite only around 32,000 being produced. The main parameters of the vehicle are shown in Table 1. The 47 kW power output may seem low, but the electric drive’s characteristics and low weight make it dynamic to drive [25].

### 2.3. Data Logging and Devices

**Figure 1:**
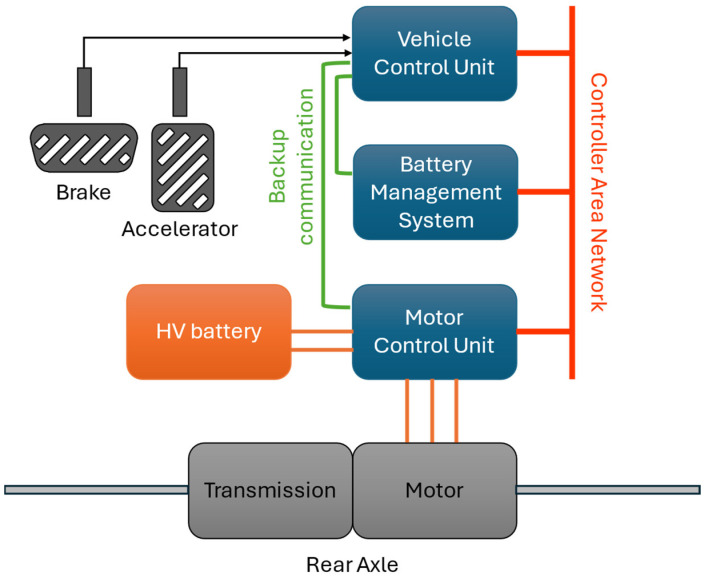
The communication network of the test vehicle.

As can be seen in Figure 1, the components of the test vehicle’s powertrain communicate with each other via the CAN bus so that the relevant parameters can be retrieved via the OBD II connector using the KWP diagnostic protocol [26].

A CSS CANedge 1 [27] standalone CAN bus data logger was used for data logging, for which the software was written by ourselves. Since gradient resistance has a major impact on consumption, the route must also be recorded. For this purpose, a u-blox NEO-M9V sensor module was used for the data logger, which has an accuracy of 2 m circular error probability and a sampling rate of 5 Hz.

The inputs of the vehicle model detailed in Section 3 are the parameters that the driver has direct control over; these are the accelerator, brake pedal, and steering wheel. Considering the additional driving resistance forces, the curvature resistance is negligible compared to the road resistance, so in this model, the curvature is not considered.

For data logging, it is important to note that for CAN 2.0, communication messages can be up to 8 bytes long, of which the diagnostics reserve 2–3 bytes as special values. In the case of KWP, typically, a local identifier can be used to retrieve data for an entire group of components, so the vehicle can send multiple response messages for a single data request; this is multi-frame messaging. However, in most cases, the sensor values are reduced to as few bytes as possible. For example, vehicle speed can be defined as an integer in 1 byte, from 0 to 255. In this example, the resolution of the speed is 1, since the minimum value change is 1. The SoC value can be from 0% up to 100%, so from 0 to 200 can be stored on 1 byte, and the minimum value difference, i.e., resolution, can be 0.5%.

The accelerator and brake pedals can be used to obtain the desired speed, but the test car’s exact pedal characteristics were unknown, so the vehicle speed was used as a reference signal. The speed can be extracted from the test vehicle with a resolution of 1 km/h, so—taking advantage of the fixed-ratio non-disconnectable drive—the speed calculated from the engine speed was used instead of the vehicle speed, which has a much higher resolution converted back to km/h. The data retrieved from the vehicle are given in Table 2.

The status of the state of charge was also monitored to investigate energy consumption. Similar to the speed, the resolution of the charge is not sufficient because the minimum value change for the test car is 0.5%. Therefore, the current and voltage of the high-voltage battery were also retrieved from the CAN bus.

## 3. Model Structure

The measurement system development aims to verify and validate a longitudinal vehicle and powertrain model that can present the energetics of a battery electric vehicle (BEV). A new approach can be introduced in BEVs because there are three in-vehicle main onboard energy storage systems: the battery, the kinetic energy of the vehicle, and the gravitational potential energy of the vehicle. The novelty of these vehicles is that the energies can be converted back and forth in any way.

### 3.1. Vehicle Model

The motion Equation (1) of the vehicle represents the sum of the forces acting on the vehicle [28], considering the rolling weights with λ. The traction, the brake, and the resistances can change the vehicle’s speed.

(1) $$\lambda m_{veh}a_{veh}=F_{trac}-F_{brake}-F_{res}.$$

Due to the regenerative charging in electric vehicles, the traction force can be negative. Thus, the brake force, which is a loss, has a minor role in vehicles with combustion engines.

The resistance forces can be handled as follows: (2) They are the sum of the road and the drag resistances.

(2) $$F_{res}=F_{road}+F_{drag}.$$

Furthermore, road resistance has two parts: rolling and grade resistance, where *α* is the road grade and *f* is the rolling resistance factor:

(3) $$F_{road}=F_{roll}+F_{grade}=m_{veh}g\left(\sin\alpha +f\cos\alpha \right)$$

The drag resistance can be handled in the following well-known way:

(4) $$F_{drag}=\frac{1}{2}\rho_{air}c_{w}A_{veh}v_{veh}^{2}.$$

The vehicle also has a conventional hydraulic brake system. Ignoring the geometrical parameters of the wheel brake systems, the brake force can be modeled as a function of the brake pressure multiplied by a constant coefficient:

(5) $$F_{brake}=k_{brake}p_{brake}mg.$$

Due to the dual braking opportunity, the brake control should have a strategy:

- The regenerative braking does not operate below 10 km/h;
- The hydraulic brake system does not generate force on the stopped vehicle;
- In battery electric vehicles, during emergency braking, the regenerative braking also does not operate; finally, this function was unnecessary because emergency braking was not tested.

### 3.2. Powertrain Model

In a BEV, the powertrain system has two parts: an electric machine and a gearbox. Due to regenerative braking, the energy flow can go back and forth in them. The losses always decrease energy flow. Thus, there are two forms of the equations.

The connection between the electric machine’s torque and the traction force on the wheels can be estimated in the following way, with positive torque:

(6) $$F_{trac}=M_{mot}\eta_{mech}\frac{i_{pt}}{r_{wheel}}.$$

During regenerative braking, mechanical efficiency should be applied with its reciprocal:

(7) $$F_{trac}=M_{mot}\frac{1}{\eta_{mech}}\frac{i_{pt}}{r_{wheel}}.$$

The mechanical efficiency represents the mechanical losses in the gearbox. In this paper, it is modeled as a constant. Without measurements, it can be estimated with the following equation [28]:

(8) $$\eta_{mech}=0.98^{a}0.97^{b}0.99^{c}.$$

A throttle input controls the actual torque of the electric machine:

(9) $$M_{mot}=u_{throttle}{Mmot}_{max}$$

This throttle with a negative sign can also operate as an input for regenerative braking. The power consumption of the electric motor can be estimated by considering its efficiency map. The equations are also different with positive and negative torque. With positive torque:

(10) $$P_{mot}=M_{mot}\omega_{mot}\frac{1}{\eta_{mot}(M_{mot},\omega_{mot})}.$$

And with negative torque, i.e., with regenerative braking:

(11) $$P_{mot}=M_{mot}\omega_{mot}\eta_{mot}(M_{mot},\omega_{mot}).$$

The model does not consider the actual temperature of the electric machine. This would change its torque characteristics. Based on the measurements, it was not necessary to implement them into the model.

### 3.3. Inverter and Battery Model

The equation logic of the energy flow through the inverter is similar to the previous ones: the losses always decrease. Up-to-date inverters have high efficiencies. In this model, it is estimated as a constant.

With positive engine torque, it can be estimated as follows:

(12) $$P_{inv}=P_{mot}\frac{1}{\eta_{inv}}.$$

Also, with negative engine torque, it is changed in the following way:

(13) $$P_{inv}=P_{mot}\eta_{inv}.$$

The current consumption of the inverter can be calculated by the battery voltage:

(14) $$I_{inv}=\frac{P_{inv}}{U_{bat}}.$$

The battery’s main parameter is its state of charge. It can be estimated by the following equation [8]:

(15) $$SoC=\frac{C_{0}-\int_{0}^{t}P_{inv}(t)dt}{C_{0}}.$$

The model does not consider the battery’s SoC-dependent charging behavior. The measurements did not start with a fully charged battery.

Other major in-vehicle energy consumers near the powertrain system include cabin heating/cooling systems, power steering systems, and other onboard electric consumers. The energy consumption of these systems can be at a wide interval. The heating/cooling system was turned off during the measurements, and only the necessary current consumers were used. Thus, the power demand of these systems was estimated to be constant.

### 3.4. Model Inputs and Driver Model

The longitudinal vehicle-end powertrain model has the following inputs:

- Vehicle speed demand, Matlab
- Hydraulic brake system actual pressure,
- Road grade.

The model can be calibrated and validated by the following signals:

- Electric motor’s actual torque and actual speed,
- Inverter actual current consumption,
- Battery’s actual voltage and state of charge.

Matlab Simulink’s (version 2023b) longitudinal driver model was applied from the Powertrain Blockset in the control loop. It was set as a PI driver.

**Figure 2:**
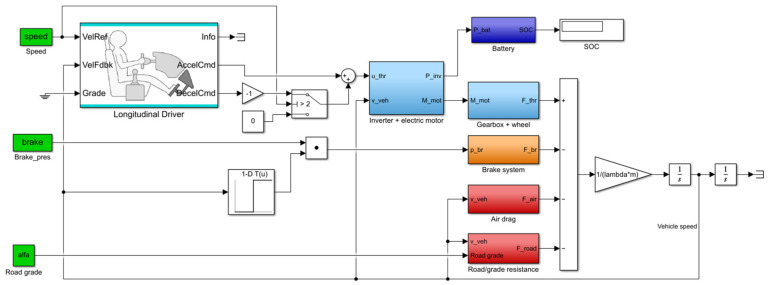
The block scheme of the longitudinal vehicle and powertrain model.

For the logically right operation, some complement functions have to be implemented into the model. At the same time, there should not be throttling or mechanical braking. Additionally, below 10 km/h, there should not be any regenerative braking. The block scheme of the model can be seen in Figure 2.

## 4. Measurements

For the model development, three routes were selected in Budapest. All of them are city roads, i.e., the maximum legal speed is 50 km/h. However, thanks to Budapest’s topography, smaller and higher road grades can be selected. The vehicle’s high load and regenerative braking can be analyzed by these.

**Figure 3:**
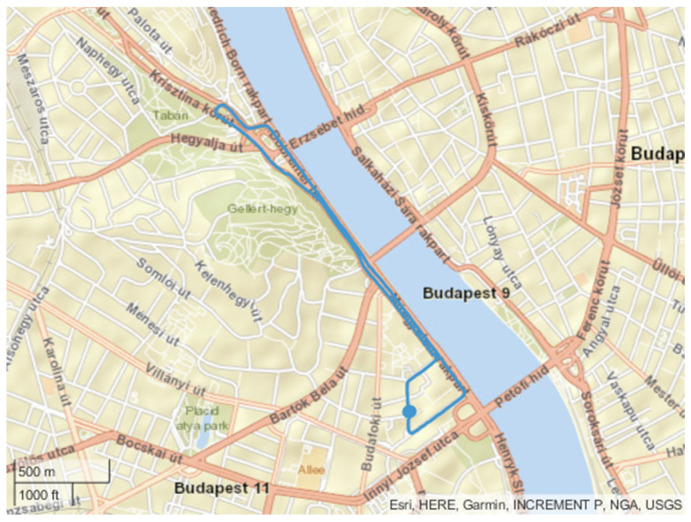
The route of the “Erzsébet” cycle.

The first applied route is called the “Erzsébet” cycle because it is between the BME University and the Erzsébet bridge along the river Duna. The route does not contain high elevation changes. The road grade changes are nearly negligible. The length is 4.7 km, and the maximum road grade is below 4%. Due to the traffic control lights, the cycle has several stops. The map of the route can be seen in Figure 3.

**Figure 4:**
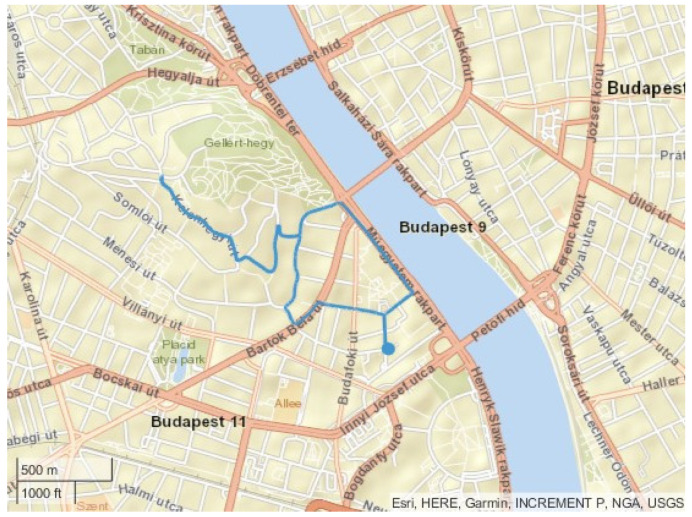
The route of the “Citadella” cycle.

The second route is called the “Citadella” cycle; the route connects the university and the Citadella castle on the top of Szent Gellért hill. The elevation change is 91 m, and the maximum road grade is 12%. The length is 5 km. The vehicle runs to the top of the cycle and then partially returns. Therefore, the cycle contains a higher load, as does the recuperation part. The map of the route can be seen in Figure 4.

**Figure 5:**
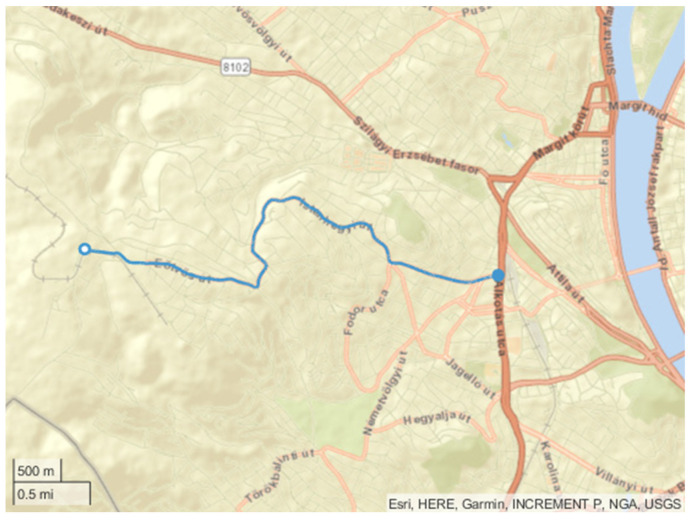
The route of the “Normafa” cycle.

The third and fourth routes are called “Normafa Up” and “Normafa Down”. Normafa is one of Budapest’s highest points. In “Normafa Up”, the vehicle is driven to the hill. The way back on the same route is “Normafa Down”. The altitude change is 326 m, and the maximum road grade is 11%. The length is nearly 6 km. The map of the route can be seen in Figure 5.

**Figure 6:**
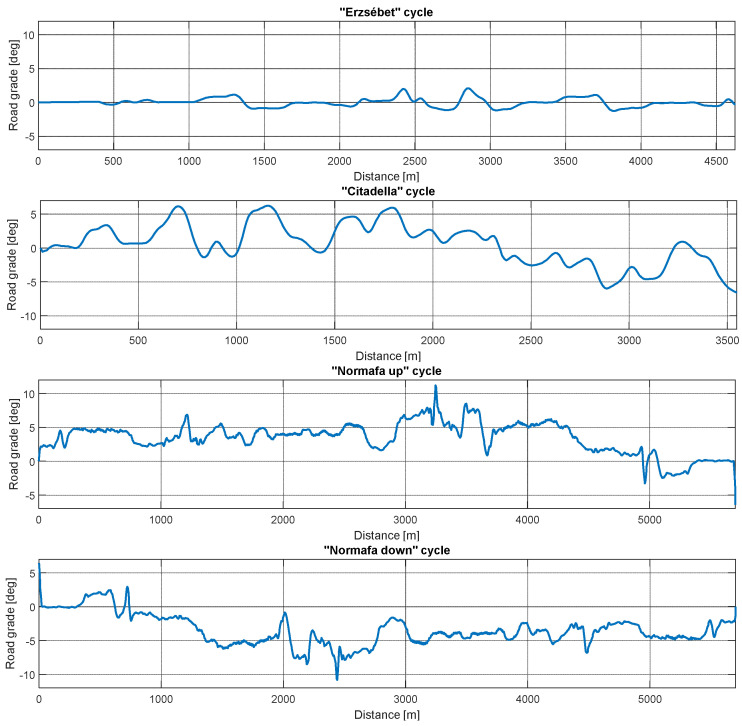
Road grade changes on the selected routes.

The road grade differences between the three routes are depicted in Figure 6. Moreover, Table 3 summarizes the main properties of the selected routes.

Several data were recorded during the measurements. For the longitudinal EV model validation, the following signals are necessary in function of time:

- High-voltage battery state-of-charge (SoC),
- Inverter input power (the voltage and the current can also be measured),
- Motor speed,
- Motor torque, which is an estimated value by the ECU,
- Vehicle speed,
- Hydraulic brake pressure,
- Route’s longitude, latitude, and altitude coordinates for estimating the elevation.

**Figure 7:**
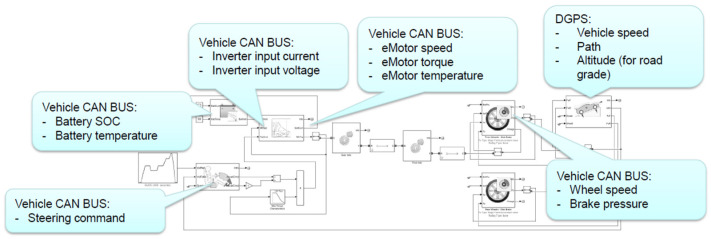
The recorded data during the measurements.

Figure 7 shows the recorded data during the measurements. In further research, the vehicle’s thermal and lateral behavior can also be analyzed.

The pressure measurement of the hydraulic brake is a key moment in the model validation; it makes it easier. Electric vehicles at slow speeds (typically below 10 km/h) do not use recuperative braking due to weak motor efficiency. In this speed range, the conventional brake system does all the braking. During the deceleration, a pre-defined strategy gradually switches between the two braking possibilities. This strategy can be analyzed by the measurements, but it is not the aim of this research. By recording the brake pressure, the braking force on the vehicle can be estimated, and the driver controller should only complement it (if necessary) by the drivetrain.

In electric vehicles, while it stays and there are no pedal commands, there is some traction torque on the drivetrain. It can be ceased by putting the gear selector on neutral or pushing the brake pedal harder This behavior should have been applied during the measurements for more accessible model validation.

## 5. Model Verification and Validation Strategy

For the first approach, most parameters can be set by the literature. The most uncertain parameters are the rolling resistance factor, the frontal area, the rotational mass factor, and the efficiency maps. With enough measurement results, these parameters can be more accurate: rolling resistance can be determined at low speeds, and air drag parameters can be set at higher vehicle speeds. Electric vehicles typically have a one-speed gearbox; its mechanical efficiency can be estimated by the number of connected gear pairs [28].

The efficiency map of the electric motor is unknown. However, the efficiency map of synchronous electric motors is not as varied as it is for internal combustion engines [29]. For this aim, the efficiency map of the first-generation Nissan Leaf’s motor characteristics [30] is used with smaller modifications: changing the speed, torque, and power range. Furthermore, smaller tuning was necessary at higher speeds and in the recuperation zone.

The wind magnitude and direction, as well as the road surface changes, are neglected in the model due to measuring issues. The measurements were made in September in good and calm weather. The most significant uncertainty is the road grade estimation. The altitudes are known from the DGPS recordings, but their accuracy is only 1 m. The data should be filtered (smoothed), and linear interpolation was used between the equidistant distances. In the presented model, the distance between two road grade points is 10 m to avoid high oscillation.

The driver model is a PI controller based on Matlab’s built-in driver controller [31]. Validation criteria can be defined for speed tracking. The root mean square (RMS) error was selected for this purpose. The aim is to keep them below 10% [32].

(16) $$RMS=\sqrt{\frac{1}{T}\int_{0}^{T}{\left(\frac{x_{meas}-x_{sim}}{x_{meas}}\right)}^{2}dt}.$$

Tuning the P and I parameters of the driver model typically results in two things. With strict requirements for speed tracking, the controller will give us nervous throttle and brake commands, i.e., the torque signal cannot be validated due to the high peak values. The torque maximums can be decreased by reducing the strict speed track requirements with lower P and I parameters in the controller. In this case, the speed track will have a small delay, as will the torque input. Thus, the torque tracking cannot be validated by the RMS error. The case of the inverter input power is the same. After several iterations, the *p* value was selected at 10, and the I value was selected at 3. In further research, more complex driver controllers can be tested; however, it is not the aim of this paper—for parameter identification, the PI controller is the right choice [33].

Considering that the modeling aim is an energetic analysis of the electric vehicle, another validation criterion can be the relative tolerance of the cumulative input energy of the inverter, i.e., the consumed energy during the cycle:

(17) $$x_{rel\_err}=\frac{\int_{0}^{T}x_{sim}dt-\int_{0}^{T}x_{meas}dt}{\int_{0}^{T}x_{meas}dt}.$$

The validation criteria can also be to keep this value below 10%.

The measured signals are used to check the energy transformation in three places in the chain:

- Vehicle speed,
- Electric motor output: speed and torque,
- Inverter input: power, voltage, and current,
- Battery SoC.

The model calibration and validation processes can be simplified by dividing the model along these chain links. For example, the electric motor’s efficiency can be checked by its inputs and outputs without the driver and drivetrain models.

Considering these, the parameter calibration was conducted using the following steps:

- Battery nominal capacity calibration is conducted using the inverter input power. Additionally, the non-drivetrain consumer’s consumption can also be set at a constant value.
- Electric motor efficiency map calibration is conducted using the input of the inverter and the motor output power (speed and torque). The inverter efficiency is estimated by a constant.
- Vehicle parameter calibration with the measured torque input. The realizable vehicle speed response should fit the measured vehicle speed. Without feedback and driver control, perfect fitting cannot be achieved due to disturbances and uncertainties. However, for parameter calibration, it is the most helpful step because the controller’s behavior can be neglected.

After these steps, the feedback model can be tested and finalized.

## 6. Model Calibration and Results

The achieved model parameters after the validation process are presented in Table 4. Most of them are in the typical range for a passenger vehicle. The rolling resistance factor is slightly lower than its usual value, probably caused by the very narrow wheels. The parameter nomenclature presents the changing parameters during the simulation in Appendix A.

The rotational mass factor is another important parameter that is not easily achievable. Its value is slightly higher than that of conventional (thermal engine) vehicles. The cause can be the different properties of electric drivetrains.

**Figure 8:**
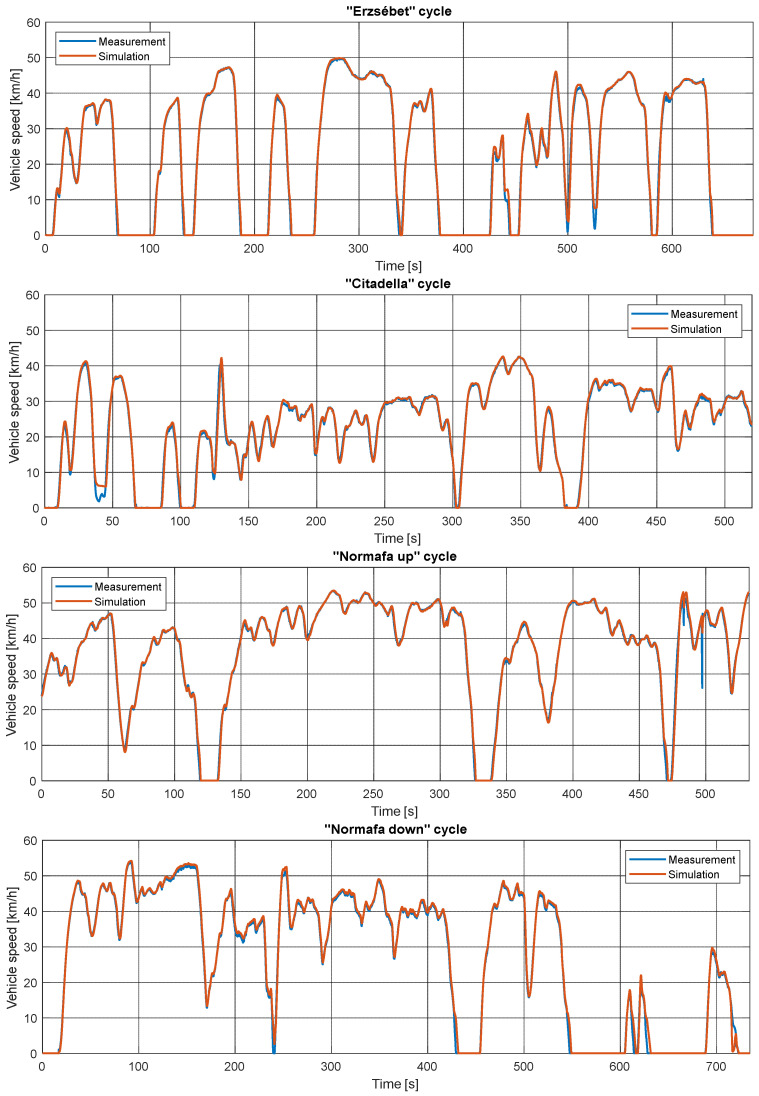
Measured and simulated speed profiles and speed tracking of the different cycles.

Figure 8 depicts the different cycles’ speed profiles and speed tracking. As can be seen, the “Erzsébet” cycle is typical city driving, with long stays due to the traffic lights. It reaches only once the legal maximum speed. Thus, the dominant property of this cycle are the slow accelerations and decelerations. The motor load is low due to the small road gradients.

The “Citadella” cycle has a smaller rate of staying. It runs through a small-speed residential area, where the legal maximum speed is sometimes 30 km/h. The narrow lanes and the high road grade resulted in a smaller speed cycle with higher motor loads.

Finally, the “Normafa” cycle’s speed results can be seen in Figure 8. For most of the cycle, the legal maximum speed is reachable, and traffic lights do not stop the vehicle too often. “Normafa Up” requires higher motor torques; “Normafa Down” provides more recuperation. It is interesting that “Normafa Down” has lower speeds.

The speed tracking can also be seen in the figure. The biggest differences are during braking. Furthermore, there are some overshoots during every acceleration.

In the following figures, the verification will be demonstrated through examples. The other omitted diagrams can be found in the Appendix B and Appendix C.

**Figure 9:**
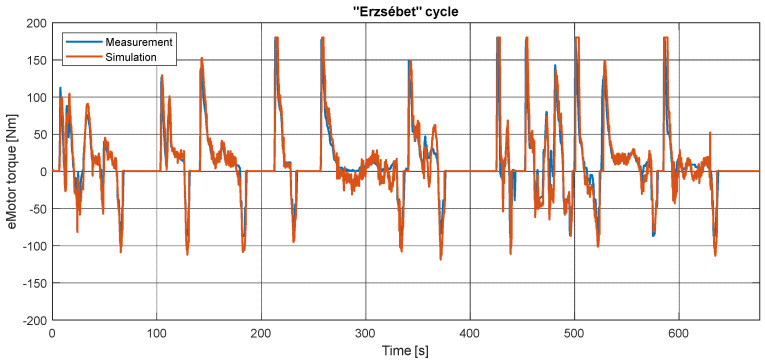
Measured and simulated torque of the electric motor during the “Erzsébet” cycle.

Figure 9 shows the measured and simulated torque during the “Erzsébet” cycle. The signal tracking is verifiable; only the peak values have some inaccuracy. It is caused by the PI driver controller’s reaction to the slight speed delay. Thus, the controller’s behavior is more nervous than that of the real driver. In the other measurements, the magnitude is different due to the altitude changes: more significant for positive road grade, more regenerative torque for negative road grade. These results can be seen in Appendix B and Appendix C.

**Figure 10:**
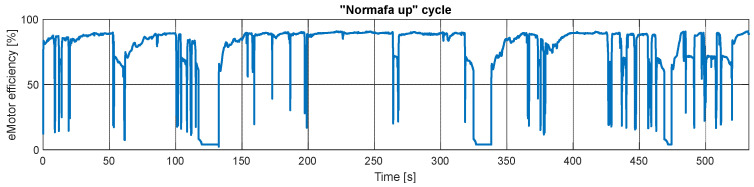
Measured and simulated efficiency of the electric motor during the “Erzsébet” cycle.

The vehicle has a permanent magnet synchronous motor, which usually has more than 95% top efficiency. In everyday use, it rarely operates at its maximum efficiency; Figure 10 shows the simulation results for the “Normafa Up” cycle. While the motor provides significant torque, its efficiency is never less than 60%. The typical magnitude is between 80 and 90%. During recuperation, it is slightly lower.

**Figure 11:**
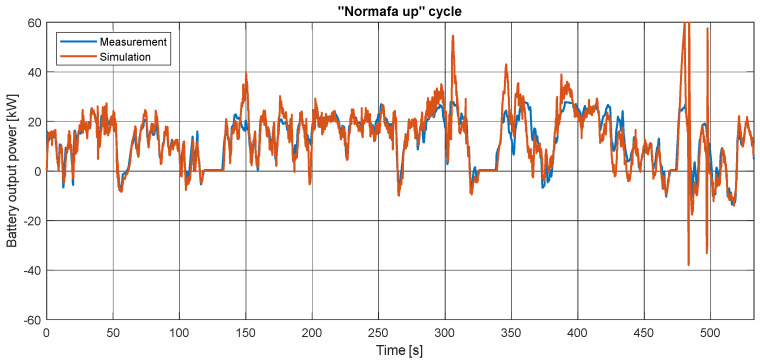
Measured and simulated battery output power during the “Normafa” cycle.

In the same measurement, the battery output power is depicted in Figure 11. The behavior is like the torque’s: it is verifiable but has inaccurate peaks and a nervous oscillation. At the last phase of the cycle, while the driving style is more aggressive, this inaccuracy increases. As earlier, the other results can be seen in Appendix C; they have similar properties.

The validation criteria can be checked using the simulated speed and inverter input power results. After the iterative model development, Table 5. contains the values they achieved. All of them are below 10%, i.e., the model is acceptable for powertrain energetics analysis.

The energetic analysis is presented in Table 6. The first three cycles of the four are energy consumers, and the fourth one recuperates more energy than it consumes. Thus, the input energy in the first three cycles is from the battery (inverter input energy). In contrast, in the fourth cycle, road resistance works as an energy source due to the decreasing altitude.

In the “Erzsébet” cycle, the vehicle runs with low loads. This case does not use the electric motor’s good efficiency zones. Thus, nearly half of the invested energy becomes motor losses. In this usage, the gearbox’s losses also occur at a significantly high rate. Due to the low speeds, the air drag does the least work. During the cycle, the recuperated energy is 62% of the traction work, nearly six times higher than the braking work.

In the “Citadella” cycle, the motor load is higher: in the first 2/3, the vehicle runs up to the hill, and then it partially runs down. In these higher loads, the motor’s efficiency is improved, and the losses decreased to 38.3%. In contrast, the road resistance work increases to 35.3%.

In the “Normafa Up” cycle, the traction work is much higher than the recuperated energy due to the high altitude increase. In this high-load motor operation, the electric motor losses decrease to 18%. Nearly 70% of the invested energy is used to overcome road resistance.

In the “Normafa Down” cycle, the behavior is the opposite. Due to the decrease in altitude, the vehicle’s potential energy transforms into losses and recuperated energy. More than 46% of the “invested” energy was recoverable in this cycle. The recuperative braking motor efficiency is lower; thus, motor losses are more than 35%. Additionally, braking loss also increases to 9%. Recuperated energy is more than six times higher than traction work.

**Figure 12:**
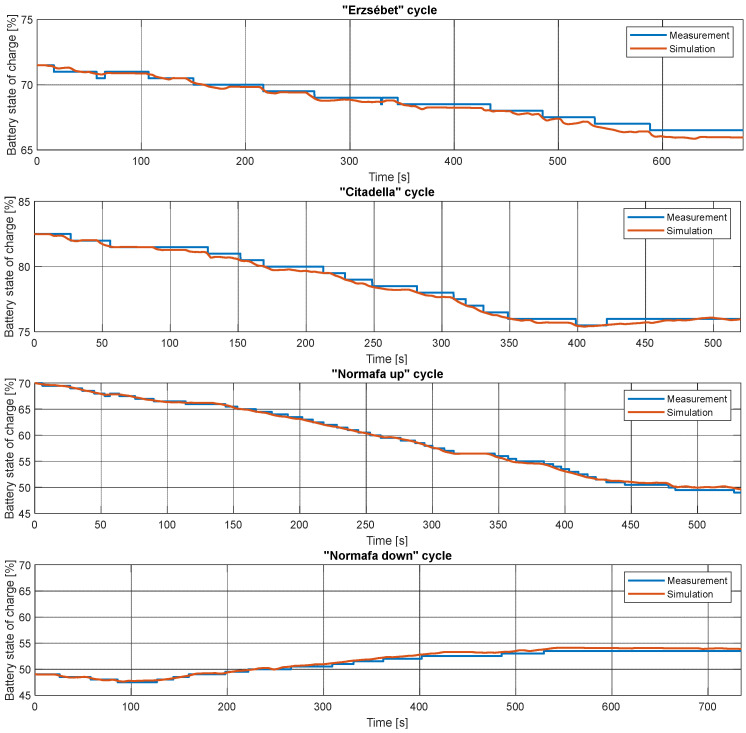
High-voltage batteries measured and simulated SoC changes during the cycles.

Finally, the high-voltage battery’s measured and simulated SoC changes in the different cycles are shown in Figure 12. The results were not used for validation because the battery SoC estimation of the vehicle should not necessarily be linear, as its characteristics are unknown. For instance, it can depend on the actual driving style. Apart from that, the diagrams demonstrate that the model can be calibrated to follow this signal accurately.

## 7. Conclusions

This paper aims to present a measurement system for BEV modeling and analysis. These vehicles’ energy management, recuperation strategy, and range prediction are important research topics. The paper provides a solution to quickly process the real-world driving cycle’s data and analyze it with simulation tools. The primary outcomes are the experiences with the measurement system and the model calibration. This paper provides the necessary signals for the analysis and gives suggestions for the sample time, too.

Due to the recuperation opportunity, knowing the road grade is particularly important. The only costly part of the measurement system is a GPS system that can record the actual altitude of the vehicle. It can be substituted with, for example, Google-based data, but its accuracy probably will not be enough. The simulation experiences confirmed that road grade accuracy is crucial for the model’s accuracy.

This paper focuses on city driving with higher altitude changes. The simulation model is a longitudinal vehicle-drivetrain model investigating the energy transformation from the high-voltage battery to the vehicle’s motion. Some typically hard-to-reach data also become estimable by the measurements, such as the additional rotational masses or the rolling resistance factor. The degradation of the battery can also be detected by the energy consumption and the HV battery SoC changes—in our case, the implemented nominal battery capacity is also significantly lower than the official data.

The presented measurement system can be the basis for several research studies on BEVs. The driving styles can also be analyzed by recording to develop the pedal characteristics and recuperation strategies for better vehicle efficiency. Additionally, by monitoring the motor and battery temperature signals, the thermal behavior can also be analyzed, and more complex battery models can be implemented. Furthermore, we also recorded the steering angle to investigate the lateral dynamics of the vehicle.

## Figures and Tables

**Table 1:** Parameters of the test vehicles.

Main Group	Parameter	Value
Body	Dimensions (L × W × H)	3395 × 1475 × 1610 mm
	Mass	1100 kg
	Propulsion type	Rear wheel drive
Motor	Type	PMSM
	Max. power	47 kW
	Max. torque	180 Nm
	Transmission	6.11
Battery	Type	Lithium-ion
	Nominal voltage	330 V
	Max. output power	60 kW
	Nominal/Useable capacity	16 kWh/14.5 kWh
Range	EPA	95 km
	NEDC	160 km
Resistance	Drag coefficient	0.33
	Drag area	2.14 m^2^
	Rolling resistance	0.011

**Table 2:** List of logged parameters with the smallest value change.

Parameter	Unit	Resolution
Speed	km/h	1 km/h
Engine speed	RPM	1 RPM
Brake system pressure	Bar	0.0154 Bar
SoC	%	0.5%
HV battery voltage	V	2 V
HV battery current	A	1 A
Torque	Nm	0.1 Nm
Height above sea level	m	1 m

**Table 3:** Main properties of the selected routes.

	“Erzsébet” Cycle	“Citadella” Cycle	“Normafa” Cycle
**Type**	essentially flat road	uphill then downhill	same route twice: up and down
**Distance**	4.7 km	5 km	5.8 km
**Time**	~12 min	~17 min	up ~9 min, down ~12 min
**Road grade**	maximum 4%	maximum 12%	maximum 11%

**Table 4:** The values of the calibrated model parameters.

Name	Sign	Value	Unit
Air density (20 °C)	*ρ_air_*	1.188	kg/m^3^
Battery nominal capacity	*C_0_*	10	kWh
Brake force coefficient	*k_brake_*	0.012	1/bar
Drag coefficient	*c_w_*	0.25	-
Electric machine’s maximum power	*P_max_*	45	kW
Electric machine’s maximum speed	*n_max_*	10,000	1/min
Electric machine’s maximum torque	*M_max_*	180	Nm
Gravitational acceleration	*g*	9.81	m/s^2^
Inverter efficiency	*η_inv_*	0.99	-
Number of loaded axle joints	*c*	2	-
Number of loaded bevel gears	*b*	0	-
Number of loaded spur gears	*a*	2	-
Road grade	*α*	0	°
Rolling resistance factor	*f*	0.005	-
Rotational mass factor	*λ*	1.2	-
Transmission gear ratio	*i_pt_*	6.11	-
Transmission mechanical efficiency	*η_mech_*	0.97	-
Vehicle frontal area	*A*	2	m^2^
Vehicle mass	*m_veh_*	1300	kg
Wheel radius	*r_wheel_*	0.2784	m

**Table 5:** RMS errors and cumulative relative errors for validation checking.

	“Erzsébet” Cycle		“Citadella” Cycle		“Normafa Up” Cycle		“Normafa Down” Cycle	
Speed validation RMS error	6.337	%	5.076	%	3.052	%	6.792	%
Inverter input power cumulative relative error	7.449	%	2.902	%	1.956	%	1.567	%

**Table 6:** Energetic analysis of the pre-defined cycles.

	“Erzsébet” Cycle		“Citadella” Cycle		“Normafa Up” Cycle		“Normafa Down” Cycle	
	kWh	%	kWh	%	kWh	%	kWh	%
Inverter input power	0.594	100.00	0.612	100.00	1.888	100.00	−0.5248	46.28
eMotor losses	0.292	49.11	0.234	38.30	0.339	17.96	0.404	35.63
Gearbox losses	0.086	14.47	0.051	8.34	0.125	6.60	0.0399	3.52
Braking work	0.091	15.24	0.079	12.97	0.011	0.56	0.1021	9.00
Air resistance work	0.045	7.59	0.020	3.23	0.066	3.49	0.0634	5.59
Road resistance work	0.081	13.63	0.218	35.63	1.310	69.39	−1.134	100.00
Traction work	0.570		0.543		1.573		0.1783	
Recuperated energy	0.353		0.217		0.149		1.147	

**Figure A1:**
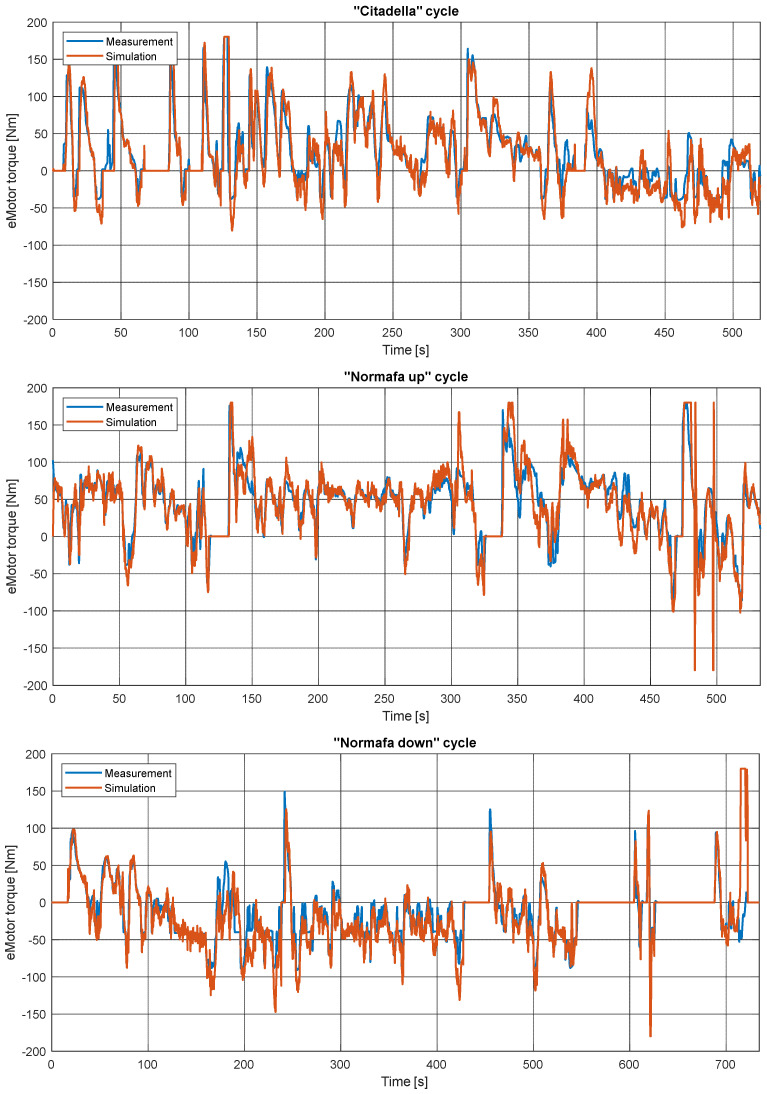
Measured and simulated the torque of the electric motor during the cycles.

**Figure A2:**
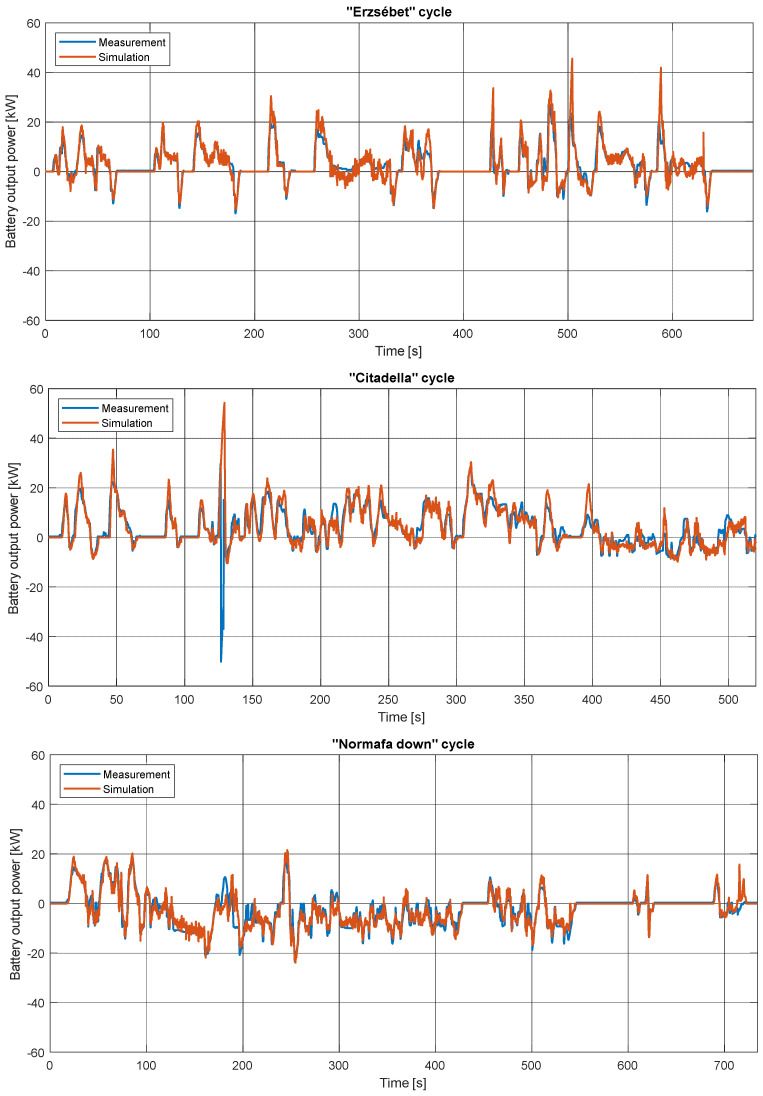
Measured and simulated battery output power during the cycles.

## Data Availability

The data presented in this study are available on request from the corresponding author.

## Appendix A

**Table A1:** Nomenclature.

**Name**	**Unit**
Battery state of charge	*SoC%*
Battery voltage	*U_bat_*
Braking force	*F_brake_*
Drag resistance	*F_drag_*
Electric machine angular velocity	*ω_mot_*
Electric machine’s efficiency map	*η_mot_*
Electric machine’s power	*P_mot_*
Electric machine’s torque	*M_mot_*
Hydraulic brake pressure	*p_brake_*
Inverter current consumption	*I_inv_*
Inverter power	*P_inv_*
Powertrain control input	*u_throttle_*
Resistance forces	*F_res_*
Road grade resistance	*F_grade_*
Rolling resistance	*F_roll_*
Traction force	*F_trac_*
Vehicle acceleration	*a_veh_*
Vehicle speed	*v_veh_*

## Appendix B

Measured and simulated motor torque results that were omitted from the discussion.

## Appendix C

Measured and simulated battery output power results that were omitted from the discussion.
